# Promoter-Operating Targeted Expression of Gene Therapy in Cancer: Current Stage and Prospect

**DOI:** 10.1016/j.omtn.2018.04.003

**Published:** 2018-04-12

**Authors:** Chao Chen, Dongxu Yue, Liangyu Lei, Hairong Wang, Jia Lu, Ya Zhou, Shiming Liu, Tao Ding, Mengmeng Guo, Lin Xu

**Affiliations:** 1 Special Key Laboratory of Gene Detection and Therapy of Guizhou Province, Guizhou 563000, China; 2 Department of Immunology, Zunyi Medical University, Guizhou 563000, China; 3 Department of Medical Physics, Zunyi Medical University, Guizhou 563000, China

**Keywords:** targeted expression, promoter, microRNAs, cancer

## Abstract

The technique of targeted expression of interesting genes, including distinct delivery systems and specific gene promoter-operating expression, is an important strategy for gene therapy against cancers. Up to now, extensive literature documented the efficacy of distinct delivery systems, such as the liposome system, nano-particle system, polyetherimide (PEI) system, and so on, in cancer gene therapy. However, a related document on the potential value of using a specific gene promoter, such as a tumor suppressor, in cancer gene therapy was still scary. The main obstacle might be that the selection of an ideal gene promoter to operate interesting gene expression in cancer gene therapy is still not fully understood. Therefore, many efforts need to be done in order to make it a real power tool for the human clinical treatment of cancer patients. The purpose of this review is to clarify the current state and some problematics in development of promoter-operating targeted expression of interesting genes and highlight its potential in cancer gene therapy.

## Main Text

Over time, the incidence of life-threatening tumors gradually increased; however, the mechanisms involved in tumor development have not been fully elucidated. The overall 5-year survival rate for cancer patients with surgery, chemotherapy, radiation, and other conventional treatments is still very low. Thus, the development of safe and effective alternative treatment strategies has become a major goal for researchers worldwide to improve cancer clinical outcomes.

Gene therapy, which has developed with the maturity of DNA recombination technology and gene cloning technology, is one of the most revolutionary medical technologies. It is to change the human genetic-material-based biomedical treatment and shows a unique advantage in the treatment of major diseases.^1^, ^2^ The history of gene therapy dates back to 1968; Rogers and Pfuderer^3^ first put forward the concept of gene therapy in *Nature*, which was documented as “use of viruses as carriers of added genetic information.” Up to now, gene therapy mainly has referred to the use of molecular biology methods to transfer human normal genes or therapeutically interesting genes through a certain way into human target cells or tissues, to correct gene defects or play a role in treatment, so as to achieve ideal outcome of modern biomedical treatment of diseases.^4^ With the development of biotechnology, gene therapy has been extended to treat malignant tumors, cardiovascular diseases, autoimmune diseases, metabolic diseases, and other critical diseases from the treatment of single-gene inherited diseases. Among them, the clinical trials of gene therapy for various malignancies accounted for two-thirds of the total. In 2004, the world’s first listed gene therapy drug was also restrictedly applied at cancer therapy, showing that tumor gene therapy was the most active and important research field of gene therapy.^5^

**Figure 1:**
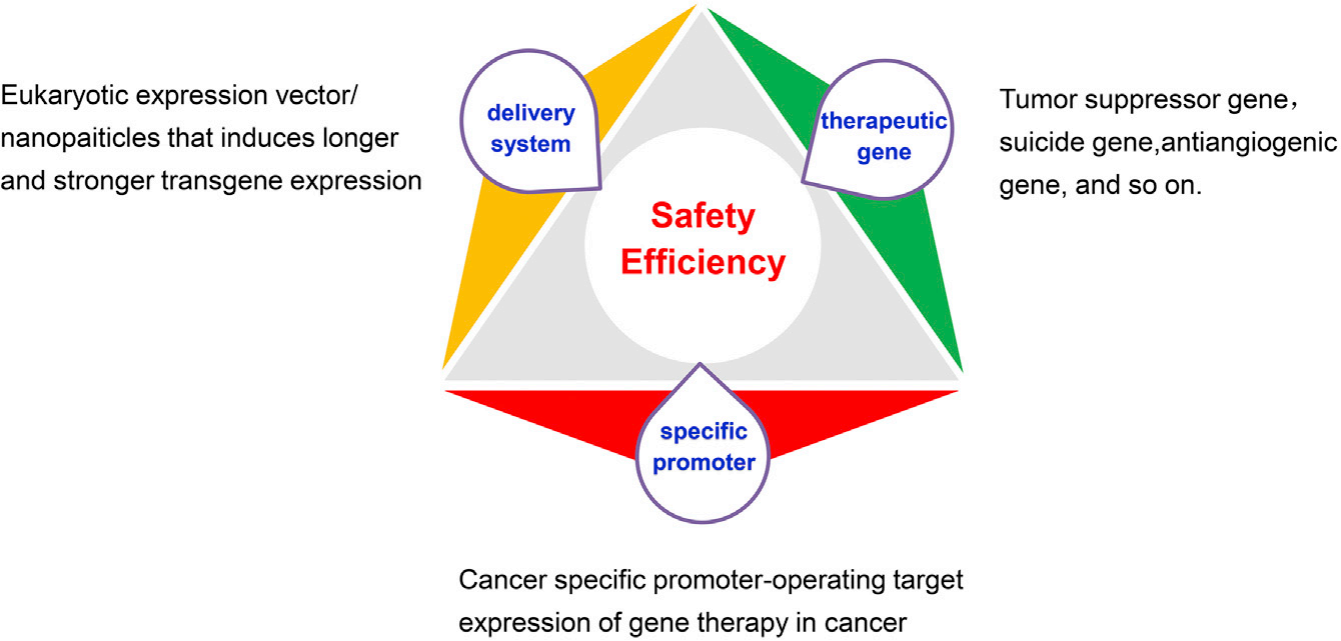
A Sketch of Targeted Expression of Genes Operated by Tumor-Specific Promoter

With the deep study of the mechanism of tumor development, people have realized that the tumor is a kind of genetic disease. Therefore, people have high expectations for gene therapy against tumors. After decades of development, gene-therapy-related technologies have matured, and several key technologies have been breakthroughs. Therefore, the next few years will be the key period of the development of cancer gene therapy. However, there are still many problems that would be effectively solved for the application of gene therapy in cancer. Among them, the key factors in cancer gene therapy are the targeting efficiency and the safety, that is to say the new therapeutic agents should specifically act on tumor cells, meanwhile reducing the damage to the normal cells. More recently, evidence suggested that the technique of targeted gene expression, including specific gene promoter-operating expression and diverse delivery systems, could better resolve targeting efficiency and safety problems, which are the key issues for the application of gene therapy for various cancers (Figure 1).

In this review, we summarize current improvements made in promoter-operating targeted expression of gene therapy in cancer. Moreover, at the end of the article, we discuss challenges and future perspectives of promoter-operating targeted gene expression in cancer gene therapy.

### The Principle of Promoter-Operating Targeted Expression of Gene Therapy in Cancer

The promoter is the upstream regulatory unit of the genetic region. Each gene has a promoter sequence at the 5′ phosphate terminus as a transcription start site, which is one of the important conditions for mRNA transcription to be initiated with the binding of RNA polymerase.^6^ Current studies have demonstrated that the promoter is activated by some specific transcription factors, and the promoter has tissue-specific regulatory function.^7^ Moreover, to tumor cells, there were some specific promoters that had specific transcriptional activity in tumor cells and no transcriptional activity or very low transcriptional activity in normal cells. For the moment, we termed these specific promoters as tumor-specific promoter (TSP). Thus, for cancer gene therapy, tumor-specific expression of interesting genes can be achieved by using TSPs to control these genes’ expression at the transcriptional level. In detail, these TSPs were constructed into a eukaryotic vector or virus vector to operate the expression of therapeutically interesting genes, such as tumor suppressor genes, suicide genes, antiangiogenic genes, and so on, and to archive valuable effects.

**Figure 2:**
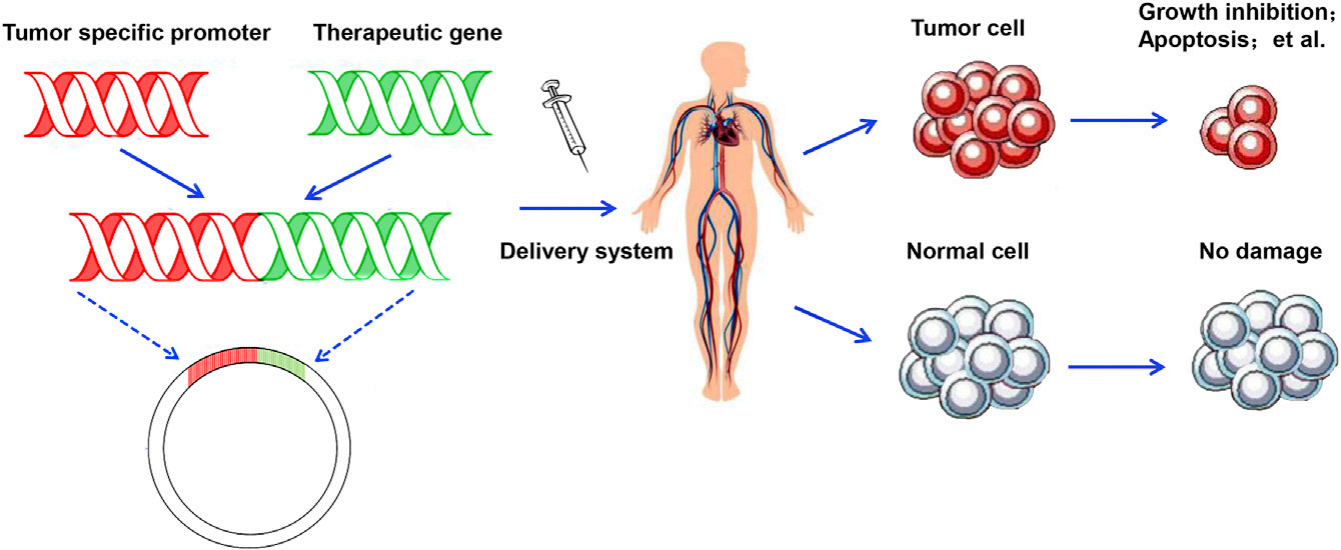
The Principle of Promoter-Operating Targeted Expression in Cancer Gene Therapy

Therefore, the critical distinct principle of promoter-operating targeted expression of gene therapy in cancer is the optimal selection of TSPs that have specific activity in target cancer cells and subsequently operated the expression of interesting genes specifically in cancer cells, accordingly avoiding the damage to normal cells (Figure 2).

### The Current Selection of Tumor-Specific Promoter in Cancer Gene Therapy

#### Promoter of Cholecystokinin Type A Receptor

Pancreatic cancer is an aggressive malignancy with morbidity rates almost equal to mortality rates because of the current lack of effective treatment options. In 2006, Li et al.^8^ reported that they modified and used the promoter of cholecystokinin type A receptor (CCKAR), which possessed relatively high activity in pancreatic cancer cells as compared with normal cells, to drive BikDD expression in a nude mouse xenograft mode of pancreatic cancer. They demonstrated an effective treatment by using the promoter of CCKAR, suggesting the feasibility of pancreatic-cancer-specific promoter-based gene therapy in pancreatic cancer treatment. Interestingly, in 2007, this research group further described a targeted approach to treat pancreatic cancer, in which they developed a versatile expression vector “VISA” operated by pancreatic-cancer-specific promoter (CCKAR-VISA), in which VP16-GAL4-WPRE was integrated with a systemic amplifier and a promoter gene of CCKAR, to target BikDD expression, a potent proapoptotic gene, in pancreatic tumors *in vivo*.^9^ Their data showed that this VISA system exhibited significant antitumor effects on pancreatic cancer and prolonged survival time of mice in multiple xenograft and syngeneic orthotopic murine models of pancreatic tumors, including the enhanced average tumor-specific CCKAR promoter activity of 600–700 times and the increased gene therapy specificity index, as well as prolonged expression time of 3–4 times. In 2014, they tried to develop human telomerase reverse transcriptase (hTERT)-VP16-Gal4-WPRE integrated systemic amplifier composite (T-VISA) vector and observed that the systemic administration of AT-VISA-BikDD encapsulated in liposomes could significantly repress the growth of prostate tumor and prolong the survival time of mice in orthotopic murine models, as well as in the murine transgenic adenocarcinoma prostate model, indicating the therapeutic potential of AT-VISA-BikDD in treatment of androgen-dependent prostate cancer (ADPC) and castration-resistant prostate cancer (CRPC) in the preclinical setting.^10^ These series of interesting works suggested the value of the usage of remodeled promoter of tumor-specific surface membrane molecules in increasing the efficiency of gene therapy.

#### Promoter of Vascular Endothelial Growth Factor Receptor

Tumor cell growth depends on vascular nutrition and oxygen supply. Then, the blocking on angiogenesis in tumor tissues is one of the effective ways to kill tumor or prevent tumor metastasis. Vascular endothelial growth factor (VEGF) is highly surface expressed in various cancer cells and is the most important angiogenesis-promoting factor, which can act on the vascular endothelial cells and induce the formation of tumor blood vessels, successively supplying oxygen for tumor growth. Kinase domain insert containing receptor (KDR), one of the two high-affinity tyrosine kinase receptors of VEGF, is lowly expressed in normal vascular endothelial cells and highly expressed in tumor vascular endothelial cells.^11^ Moreover, gene-directed enzyme pro-drug systems such as herpes simplex virus thymidine kinase (HSV-TK), in combination with the pro-drug ganciclovir (GCV), are reported as the most commonly used anti-cancer gene therapy system both in experimental models and clinical trials.^12^ Interestingly, Wang et al.^13^ used the differential expression of KDR in vascular endothelial cells between tumor tissues and normal tissues, and designed a KDR promoter-based HSV-TK expression adenovirus-mediated transfer system (AdKDR-tk), which only targeted expression of HSV-TK in tumor vascular endothelial cells. Then, recombinant adenovirus AdKDR-tk was used to infect KDR-expressing vascular endothelial cells human umbilical vein endothelial cells (HUVECs) *in vitro*. Their data showed that the survival rate of HUVECs infected with AdKDR-tk decreased significantly in the presence of GCV, a pro-drug for inducing cell death. While the survival rate of HepG2 cells, without KDR expressing, infected with AdKDR-tk was not significantly affected, indicating that KDR promoter could effectively regulate the specific expression of HSV-TK in KDR expression vascular endothelial cells, which might be a potential selection for targeted gene therapy in various cancers.

#### Promoter of hTERT

Telomerase is a reverse transcriptase that can reverse the synthesis of deoxyribonucleic acid by using an RNA sequence as its template. Its main function is to add a sequence of particles at the end of the chromosome to keep the telomere length stable. The activation of telomerase leads to unlimited proliferation of cells. Telomerase activity is evidently detected in the vast majority of tumors (in >90% of human cancers), but not in most normal cells.^14^, ^15^ Moreover, activation of telomerase is tightly regulated at the transcriptional level of the hTERT. Therefore, the use of the hTERT promoter-driven vector system could restrict the expression of therapeutically interesting genes to telomerase-positive tumors.

In 2001, Koga et al.^16^ first constructed an hTERT promoter-driven vector system that restricted the expression of fas-associating protein with a novel death domain (FADD) in telomerase-positive tumor cells. Their data showed that this system (hTERT/FADD) could effectively induce the apoptosis of tumor cells and inhibit the growth of tumor cells *in vivo*.^16^ At the same time, Komata et al.^17^ also reported that the hTERT promoter-operated expression of caspase-8 could significantly induce the apoptosis in hTERT-positive malignant glioma cells, but not in hTERT-negative astrocytes, fibroblasts, and alternative lengthening of telomeres cells, and significantly suppressed the growth of malignant glioma cells in the *in vivo* experimental setting. These works strongly suggested that the telomerase-specific transfer of distinct genes under the hTERT promoter is a novel targeting approach for the treatment of cancers.

#### Promoter of Thyroid Transcription Factor-1

Thyroid transcription factor (TTF)-1 is a member of the homeodomain-containing Nkx2 family of transcription factors. Recent evidence showed that TTF-1, as a lineage-specific oncogene, was dominantly expressed in lung cancer, but not other types of cancers, and its expression level was closely correlated with the prognosis of lung cancer patients.^18^, ^19^, ^20^ MicroRNA-7 (miR-7) is a unique member of miRNAs and plays an important role in the progression of various tumors.^21^, ^22^ Our previous works showed that miR-7 overexpression could obviously reduce the growth and metastasis of human lung cancer cells *in vitro* and *in vivo*.^23^ Moreover, the reduced expression of miR-7 was associated with the site’s mutation of its promoter region in lung cancer tissues.^24^ These data suggested that miR-7 may be a promising candidate for gene therapy against lung cancer.^25^ Most recently, our group first tried to construct a eukaryotic vector of promoter of TTF-1 gene-operating expression of miR-7 (termed p-T-miR-7) and observed its effects on the growth and migration of human lung cancer cells *in vivo*. Interestingly, we found that the growth and metastasis of human lung cancer cells *in vivo* was significantly reduced in remote hypodermic injection of the p-T-miR-7 group, accompanied by increased expression of miR-7 and altered transduction of the Akt and Erk pathway *in situ* in a lung cancer xenograft model in nude mice.^26^ Thus, these data for the first time indicate that TTF-1 promoter-operating distinct miRNA molecule expression also might be an ideal strategy for targeted expression of distinct miRNAs in lung cancer and were helpful for the development of gene therapy against clinical lung cancer.

### Challenge and Future Perspectives

#### Efficiency

A large number of reports on TSPs have shown that TSPs have much lower activity than the commonly used (conventional) strong cytomegalovirus (CMV) enhancer/promoter, which is ubiquitously active without tumor specificity. Moreover, the efficiency of gene therapy depends greatly on the efficiency of interesting gene expression after systemic delivery. Therefore, one of the researchers’ goals in the current period is to develop a cancer-specific expression vector that would not only maintain cancer specificity, but also produce robust activity stronger than or comparable to that of the CMV promoter-driven expression vector in cancer cells, but much lower activity in normal cells. Notably, in 2012, Xie et al.^27^ developed a versatile T-based breast-cancer-specific promoter VISA composite (T-VISA) to target transgene expression in breast tumors and found that T-VISA has stronger activity comparable with or higher than that of the CMV promoter in cancer cells. They further found that the targeted expression of therapeutic gene BikDD driven by the T-VISA promoter could inhibit tumor cell growth at least as effectively as CMV-BikDD *in vitro* and in a dose-dependent manner. They further compared the therapeutic effects of T-VISA-BikDD and CMV-BikDD in an *in vivo* experimental setting, and confirmed that T-VISA-BikDD nanoparticles could more obviously inhibit tumor growth and prolong the survival rate of mice than CMV-BikDD nanoparticles did in the syngeneic orthotopic murine model.^27^ This interesting research suggested that optimal modification of an artificial promoter may be a useful approach to improve the target efficiency of targeted gene therapy against cancer.

#### Safety

Currently, the safety of gene therapy strategy, mainly including functional change of important organs and distribution of exogenous DNA, is another critical challenge for the potential application of promoter-operated gene expression therapy in cancer.^28^, ^29^, ^30^

In the aspect of distribution of exogenous DNA, Mahato et al.^31^ investigated the disposition characteristics of pDNA complexed with cationic liposomes after intravenous injection in mice, and found that liposomal pDNA encoding gene expression enriched in lung, heart, kidney, and spleen. Thanaketpaisarn et al.^32^ further reported that naked plasmid DNA (pDNA) encoding firefly luciferase was directly injected into the tail vein of mice, and found that the plasmid mostly enriched in liver, spleen, and kidney. Besides, some literature documented naked pDNA enriched in the liver *in vivo* after hydrodynamic injection via tail vein.^33^ Different from these works, in our recent research,^26^ we analyzed the distribution of naked plasmid p-T-miR-7 after remote hypodermic injection and found that the plasmid dominantly enriched in lung tissue and tumor mass *in vivo*. To the diverse phenomenon, we proposed two factors may be closely related to the different distribution of pDNA *in vivo*. The first factor was the different experimental setting including entry route and way, as well as the dosage, of naked pDNA. The second factor was the different hemodynamics and perfusion in distinct organs and tissues.

To the functional change of important organs, Xie et al.^27^ treated 4T1 tumor-bearing mice with the pDNA/nanoparticle complexes carrying either CMV-Luc or T-VISA-Luc via tail vein. Their data showed that T-VISA-BikDD induced remarkable cell apoptosis in tumor tissues, but not in the normal tissues such as the lung and the liver, whereas CMV-BikDD led to substantial apoptosis both in tumor tissues and in surrounding organs. To further evaluate whether treatment with T-VISA-BikDD was safer than CMV-BikDD, they used a single dose of 50 or 100 μg of pDNA to treat BALB/c mice by tail-veil injection and analyzed serum levels of alanine transaminase (ALT) and aspartate transaminase (AST). Their data showed that serum levels of ALT and AST increased on day 2 after injection in the CMV-BikDD treatment group, whereas they were within the reference range for the control in T-VISA-BikDD treatment groups at any measured time points. Similar to their finding, our recent work also showed that, after plasmid p-miR-7 subcutaneous injection treatment, there were not any significant pathological changes on both local skin tissue and some other important organs, including liver and heart, in the nude mouse model of human lung cancer.^26^ Combining these results, at least, indicated the potential safety of tumor-specific promoter-targeted expression of distinct genes in cancer gene therapy. However, the exact safety of promoter-operating targeted gene therapy, including gene integration and degradation time course, in cancer gene therapy still remains to be fully elucidated in the future.

#### Future Perspectives

In the last 20 years, there was outstanding progression on the research on promoter-operating targeted expression of gene therapy in cancer. In the future, there are still some core issues that should be further addressed, which will much benefit the ultimate application of promoter-operating targeted expression of gene therapy approach in clinical cancer patients. The first is still the optimal selection of the TSPs, which is a critical factor for specificity, efficiency, and safety problems of target gene therapy in cancer. Up to now, there have been some other alternative candidate TSPs used in different experimental studies (Table 1). However, different TSPs have their distinct advantages and disadvantages (Table 2). Therefore, it is particularly important to select the appropriate TSPs for a specific tumor. Just as we mentioned above, it is believed that the remold of artificial promoter might be a promising approach in the future, especially further verification, optimization, and modification of promoters. For example, researchers found that the adjustment of the length of promoter nucleic acid sequence is one of the optimization methods, including the verification of the core sequence of promoter.^34^ The second is the selection of more valuable interesting genes. Besides these documented interesting genes (Table 3), accumulating evidence further suggested that non-coding RNA sequences such as miRNA,^26^ short hairpin RNA (shRNA),^35^ and RNAi^36^ may be optional choices for the ideal genes in cancer gene therapy. Moreover, the identification of other new tumor-associated genes also may be helpful for the choice of interesting genes. The third is the delivery pathways and delivery system. The rational selection of drug delivery pathway will further improve the implementation of gene therapy. Moreover, the adoption of some delivery systems such as Nano carriers, with the characteristics of low toxicity, low immunogenicity, long metabolic cycle, and easy modification, are very valuable for enhancing the efficacy and reducing the potential side effect of gene therapy.^37^, ^38^ In addition, the exploration of promoter-related characteristic transcription factors, promoter epigenetic alterations, and its integrity are also helpful for the rapid development of promoter-targeted gene therapy research.

**Table 1:** The Application of Promoter in Cancer Gene Therapy

Promoters	Tumor Therapy
Promoters Targeting Non-specific Tumor Cells	
hTERT^10^, ^16^, ^17^, ^34^, ^64^	lung cancer, liver cancer, gastrointestinal cancer, breast cancer, etc.
KDR^12^, ^13^	lung cancer, liver cancer, gastrointestinal cancer, breast cancer, etc.
Bmi-1^36^	gastric cancer, prostate cancer, glioma cancer, etc.
Survivin^39^, ^40^, ^60^, ^61^, ^63^	liver cancer, gastrointestinal cancer, gallbladder cancer, etc.
HER-2^40^, ^41^	prostate cancer, breast cancer, pancreatic cancer, etc.
uPAR^42^, ^43^	colorectal cancer, colon cancer, pancreatic cancer, breast cancer, etc.
A33^44^, ^45^	colorectal cancer, gastric cancer, etc.
COX-2^44^, ^46^, ^47^	colorectal cancer, endometrial cancer, breast cancer, etc.
FGF^44^, ^48^	colorectal cancer, ovarian cancer, prostate cancer, etc.
Rad51^49^, ^50^	lung cancer, breast cancer, cervix cancer, pancreatic cancer, etc.

Promoters Targeting Specific Tumor Cells	

CCKAR^8^, ^9^	pancreatic cancer
TTF-1^26^	lung cancer
AFP^51^, ^52^	liver cancer
CEA^53^, ^54^, ^55^	gastrointestinal cancer
PSA^56^, ^57^	prostate cancer
PB^58^, ^59^, ^62^	prostate cancer

A33, glycoprotein A33; AFP, alpha fetal protein; Bmi-1, B cell-specific Moloney leukemia virus insertion site 1; CCKAR, cholecystokinin type A receptor; CEA, carcinoma embryonic antigen; COX-2, cyclooxygenase-2; FGF, fibroblast growth factor; HER-2, human epidermal growth factor receptor 2; hTERT, human telomerase reverse transcriptase; KDR, kinase domain insert containing receptor; PB, probasin; PSA, prostate-specific antigen; Rad51, rad51 recombinase; TTF-1, thyroid transcription factor-1; uPAR, urokinase-type plasminogen activator receptor.

**Table 2:** Key Features of Different Tumor-Specific Promoters in Cancer Gene Therapy

Promoters	Character	Advantages	Disadvantages
Promoter Targeting Non-specific Tumor Cells			
KDR	high activity in gastrointestinal cancer, lung cancer, liver cancer, breast cancer, etc.	tumor non-specificity, widely used for various tumors	the effect on distinct tumors may be different
hTERT	high activity in gastrointestinal cancer, lung cancer, liver cancer, breast cancer, etc.		
Bmi-1	high activity in gastric cancer, prostate cancer, glioma cancer, etc.		
Survivin	high activity in gastrointestinal cancer, lung cancer, liver cancer, breast cancer, etc.		
HER-2	high activity in prostate cancer, breast cancer, pancreatic cancer, etc.		
uPAR	high activity in colorectal cancer, pancreatic cancer, colon cancer, breast cancer, etc.		
A33	high activity in colorectal cancer, gastric cancer, etc.		
COX-2	high activity in colorectal cancer, endometrial cancer, breast cancer, etc.		
FGF	high activity in colorectal cancer, ovarian cancer, prostate cancer, etc.		
Rad51	high activity in lung cancer, breast cancer, cervix cancer, pancreatic cancer, etc.		

Promoters Targeting Specific Tumor Cells			

CCKAR	high activity in pancreatic cancer	tumor specificity, the effect on a specific tumor is relatively certain	only used for specific tumor
TTF-1	high activity in lung cancer		
AFP	high activity in liver cancer		
CEA	high activity in gastrointestinal cancer		
PSA	high activity in prostate cancer		
PB	high activity in prostate cancer		

A33, glycoprotein A33; AFP, alpha fetal protein; Bmi-1, B cell-specific Moloney leukemia virus insertion site 1; CCKAR, cholecystokinin type A receptor; CEA, carcinoma embryonic antigen; COX-2, cyclooxygenase-2; FGF, fibroblast growth factor; HER-2, human epidermal growth factor receptor 2; hTERT, human telomerase reverse transcriptase; KDR, kinase domain insert containing receptor; PB, probasin; PSA, prostate-specific antigen; Rad51, rad51 recombinase; TTF-1, thyroid transcription factor-1; uPAR, urokinase-type plasminogen activator receptor.

**Table 3:** The Application of Candidate Genes in Cancer Gene Therapy

Gene-Based Therapeutic Strategies	Candidate Genes
Suicide gene	Bik,^8^, ^9^, ^10^ HSV-TK/GCV,^12^, ^13^, ^41^, ^43^, ^46^, ^48^, ^59^ FADD,^16^ caspase-8,^17^ caspase-3,^40^ CD/5-FC,^41^, ^54^ NfsB/CB 1954,^41^, ^56^ DTA,^49^, ^50^, ^57^ tBid,^51^ TRAIL,^52^ TK/CD,^55^ Bax,^60^ etc.
Tumor suppressor gene	miR-7,^26^ E1A,^34^, ^45^ 15-PGDH,^47^ Gel,^61^ p202,^62^ P53,^63^ etc.
Antiangiogenic gene	CD105,^35^ Bmi-1,^36^ alpha3(IV)NC1^64^

alpha3(IV)NC1, noncollagenous domain of alpha3(IV) collagen; Bax, BCL2-associated X protein; Bik, Bcl-2 interacting killer; Bmi-1, B cell-specific Moloney leukemia virus insertion site 1; CD105, Endoglin; CD/5-FC, cytosine deaminase/5-fluorocytosine; DTA, diphtheria toxin A; E1A, adenoviral type 5 transcription factor that possesses anticancer properties; FADD, fas-associating protein with a novel death domain; Gel, Gelonin; p202, interferon-activated gene 202B; HSV-TK/GCV, herpes simplex virus thymidine kinase gene/ganciclovir; miR-7, microRNA-7; NfsB/CB1954, nitroreductase/5-(aziridin-1-yl)-2,4-dinitrobenzamide; 15-PGDH, 15-hydroxyprostaglandin dehydrogenase; tBid, truncated BH3 interacting domain death agonist; TRAIL, tumor necrosis factor-related apoptosis-inducing ligand.

In all, successive research on the exploration of more ideal target genes, effective promoter optimization schemes, and rational route of delivery, as well as delivery system, will undoubtedly provide a better prospect of clinical application for promoter-operating targeted expression of gene therapy in cancer.

## Author Contributions

C.C. designed and wrote the paper; D.Y. and H.W. wrote the paper; L.L., J.L., Y.Z., S.L., T.D., and M.G. analyzed the related information; L.X. conceived, designed, and wrote the paper; and all authors reviewed the paper.

## Conflicts of Interest

All authors declare that the research was conducted in the absence of any commercial or financial relationships that could be construed as a potential conflict of interest.
